# A multi-level approach to reduce exploding type 2 diabetes in Pakistan

**DOI:** 10.3389/fpubh.2025.1514090

**Published:** 2025-03-21

**Authors:** Fazal Jamil, Umaima Mir, Anum G. Niazi, Shandana Kifayat, Shanlina Kifayat, Sobia Shafiq, Zeeshan Wali, Muhammad Ali Jan Khan, Baber Wali, Khadija Tul Kobra, Muhammad Salar Khan

**Affiliations:** ^1^St. Elizabeth Youngstown Hospital, Youngstown, OH, United States; ^2^Lady Reading Hospital, Peshawar, Pakistan; ^3^Johns Hopkins Bloomberg School of Public Health, Baltimore, MD, United States; ^4^Khyber Teaching Hospital, Khyber Medical University, Peshawar, Pakistan; ^5^Lady Reading Hospital, Peshawar, Pakistan; ^6^Peshawar Dental College, Peshawar, Pakistan; ^7^Ayub Medical College, Abbottabad, Pakistan; ^8^College of Physical Medicine and Rehabilitation Paraplegic Center, Khyber Medical University, Peshawar, Pakistan; ^9^Cadet College Swat, Gulibagh, Pakistan; ^10^Islamia College University, Peshawar, Pakistan; ^11^Rochester Institute of Technology, Rochester, NY, United States

**Keywords:** type 2 diabetes, prevention measures (PM), policy interventions, Pakistan, obesity, public awareness, dietary changes, risk factors

## Abstract

Pakistan has the third-highest rate of type 2 diabetes globally, following China and India, making this a significant public health crisis. Despite the severity of the issue, efforts from health and policy practitioners to address it remain limited. With millions already diagnosed as pre-diabetic, the rising incidence of diabetes is rapidly becoming a public health emergency that demands immediate attention. This policy brief provides an accessible overview of diabetes, focusing on its types, mechanisms, and preventive measures. It also identifies key contributing factors, such as dietary habits, obesity, physical inactivity, and the influence of modern dietary trends, while proposing strategies for individuals, communities, and policymakers to combat this growing epidemic in Pakistan. The brief emphasizes the need for a multi-level approach that includes public awareness, education, behavioral and dietary changes, and policy interventions to reverse the trend. Strategies discussed include promoting healthy eating, increasing physical activity, managing obesity, and enhancing access to affordable, healthy food. Additionally, the brief highlights the importance of community and government support, such as public health campaigns, infrastructure improvements, and legislative efforts. By adopting this comprehensive approach, Pakistan can take meaningful steps to address the diabetes epidemic and improve public health outcomes.

## 1 Introduction

According to the International Diabetes Federation Diabetes Atlas, Pakistan has the third-highest rate of type 2 diabetes among adults globally, after China and India, making it a growing crisis in the country (1). Furthermore, with an estimated prevalence of 33.6% among individuals aged 20–79, Pakistan now holds the highest global ranking for type 2 diabetes (2). Despite these alarming figures, health and policy practitioners have made limited efforts to address the issue, leaving the country vulnerable to an escalating public health crisis. The situation is even more concerning, with millions already diagnosed as pre-diabetic (3). The rising incidence of diabetes is rapidly becoming a public health emergency that requires immediate attention.

To combat this epidemic, a comprehensive, multi-level approach is necessary, which would cover individual to communal and government level efforts. Our team contends that such an approach would be most effective if it included public awareness, education, outreach, behavioral and dietary changes, along with policy interventions.

This policy brief provides an accessible overview of diabetes, including its types, causes, and preventive measures. It also explores the increasing prevalence of diabetes in Pakistan, identifies contributing factors, and outlines strategies for individuals, communities, and public health authorities to effectively prevent and manage the disease within the country. As this policy brief is aimed at raising public awareness and stimulating policy and community conversations around this critical public health issue in Pakistan, we draw on relevant literature while also relying on our expertise and background to offer suggestions, which aligns with the journal’s scope for this collection.^1^ To sustain public interest and to convey our message in plain language, we have deliberately minimized the use of dense academic citations and heavy medical jargon.

## 2 Diabetes: types, mechanisms, and factors

Diabetes is a chronic medical condition marked by elevated blood sugar levels (hyperglycemia) due to impaired insulin action, insufficient insulin production, or both. Insulin, a hormone produced by the beta cells in the pancreas, facilitates the uptake of glucose from the bloodstream into muscle and other body cells for energy (4).

Among the two primary types of diabetes (type 1 and type 2), type 1 diabetes is an autoimmune disorder in which the body’s immune system destroys insulin-producing beta cells in the pancreas, leading to little or no insulin production (5). In contrast, type 2 diabetes is primarily characterized by insulin resistance and progressive pancreatic beta-cell dysfunction, resulting in relative insulin deficiency and subsequent hyperglycemia (6, 7).^2^

Hyperglycemia in diabetic patients contributes to cardiovascular complications, kidney dysfunction, and oxidative stress, as well as metabolic dysregulation, which, in turn leads to increased appetite. Several factors contribute to the development of insulin resistance in type 2 diabetes, with obesity, genetics, and environmental and lifestyle factors playing significant roles.

Obesity, particularly visceral fat, is a major contributor to insulin resistance (8). In obese individuals, adipose (fat) tissue releases various biochemical substances (cytokines and hormones) that trigger inflammation and interfere with insulin signaling pathways, leading to insulin resistance. Simply put, excess fat can disrupt normal metabolism by causing inflammation and making it harder for the body to use insulin properly.

Genetics is another determinant for insulin resistance.^3^ Studies have identified several genetic variants that predispose individuals to impaired insulin signaling, beta-cell dysfunction, and altered glucose metabolism (8). These genetic factors may explain why some individuals develop insulin resistance despite having a normal body weight, while others remain metabolically healthy despite obesity. Inherited predispositions also influence how efficiently the body processes insulin and regulates blood sugar levels, affecting the risk of developing type 2 diabetes.

Beyond genetics and obesity, insulin resistance also develops gradually due to various environmental and lifestyle factors, with dietary habits playing a significant role (9). Overconsumption of refined sugars, processed grains, and high-glycemic carbohydrates results in frequent spikes in blood sugar levels. In response, the body produces excess insulin to manage these sugar spikes. Persistent overproduction of insulin causes the body to become less responsive, or more resistant, to it. Furthermore, this ongoing cycle of elevated blood sugar and excess insulin release leads to dysfunction in insulin-producing cells, ultimately resulting in diabetes. Physical inactivity, poor sleep, and chronic stress further contribute to insulin resistance by altering hormonal balance, inflammatory responses, and glucose metabolism.

## 3 Type 2 diabetes in Pakistan: causers and mitigators

Both types of diabetes are important to consider, but in Pakistan, the focus is primarily on type 2 diabetes due to its higher prevalence and preventable nature. According to recent estimates, Pakistan has one of the highest rates of diabetes in the world, with an estimated 33 million adults living with the condition (1).

The rise in diabetes cases in Pakistan can be attributed to three major cultural changes: increased consumption of refined sugars and processed grains, reduced physical activity paired with increased screen time, and more frequent eating and snacking. Western dietary influences have introduced more sugary and processed foods into the Pakistani diet, facilitated by the rapid growth of fast-food restaurants—a trend similar to nutritional shifts seen in other low and middle income countries (LMICs) (10). Moreover, traditional staples such as high-glycemic bread and rice continue to contribute to excessive carbohydrate intake. Decreased physical activity, coupled with more sedentary lifestyles, has further exacerbated the situation by lowering insulin sensitivity and increasing fat storage.

Frequent eating and snacking on unhealthy food may also contribute to the rising incidence of diabetes in Pakistan. While the scientific link between meal frequency and insulin resistance is still being studied, some research suggests that among women who eat breakfast irregularly, meal frequency is associated with a significantly higher risk of developing type 2 diabetes (11). Other studies indicate that frequent meal may contribute to higher BMI (and, by extension, obesity) (12), both of which are major risk factors for insulin resistance.

A critical factor in Pakistan’s diabetes epidemic is central obesity, which is highly prevalent, affecting over 70% of the Pakistani population (13). Excess visceral fat not only contributes to insulin resistance but also promotes chronic inflammation, further accelerating metabolic dysfunction (14). Given this strong link between obesity and diabetes, managing weight effectively is essential to reducing diabetes risk.

Research has consistently shown that weight reduction interventions are among the most effective strategies for preventing type 2 diabetes. The Diabetes Prevention Program (DPP) and similar studies have demonstrated that even modest weight loss (5–10% of body weight) can significantly improve insulin sensitivity and reduce diabetes risk (15). Comprehensive lifestyle interventions, including caloric restriction, increased physical activity, and nutritional counseling, have proven successful in weight management and diabetes prevention efforts (16).

Beyond weight management, insulin resistance itself can be reversed through targeted lifestyle changes. These include reducing the intake of refined sugars and processed grains, focusing on whole foods with low glycemic indices, limiting snacking and incorporating intermittent fasting. While intermittent fasting primarily aids in weight loss, emerging research suggests it may also enhance metabolic flexibility and reduce inflammation (17, 18), both of which play a role in diabetes prevention. Restricting eating to an 8-h window has also been associated with lower insulin levels and improved glucose metabolism (19).

Regular physical activity is equally crucial in this effort. Exercise, particularly after meals, has been shown to improve insulin sensitivity and helps muscles absorb and utilize blood sugar more efficiently (20).

To address the diabetes epidemic in Pakistan, comprehensive public health strategies are vital. Nationwide programs should be implemented to educate the public about healthy eating, weight management, and the risks associated with refined sugars and processed foods. Promoting physical activity through community programs, as well as initiatives in schools and workplaces, is equally important. Enhancing access to affordable, healthy food options—especially in urban areas where sedentary lifestyles and processed foods are more common—can have a significant impact. Additionally, regular screenings for prediabetes and diabetes are crucial for early detection and management.

## 4 Multi-level approach

Addressing the diabetes crisis in Pakistan requires a unified effort at multiple levels, including individuals, communities, and policymakers (see Table 1 for more details). By adopting such a multi-level approach that encourages informed lifestyle changes and the implementation of effective public health strategies, we propose it is possible to reverse the trend and mitigate this growing health crisis.

**Table 1:** A multi-level approach to reduce diabetes in Pakistan.

Level	Interventions
Individual	*Screen time alternatives*: Reading, outdoor games, engaging hobbies, family activities, community recreational activities *Dietary changes*: Replacing coke with sparkling water, refined bread with whole grain or oat flour, avoiding fast food (pizza, burger, and shawarma), cutting out sugar in tea, increasing intake of non-starchy fruits and vegetables (watermelon, cucumber, orange, apple, spinach, tomatoes) and avoiding rice, replacing ghee with cooking vegetable or corn oil, choosing home-cooked meals, eating dinner before sunset (maghrib), drinking 8 glasses of water *Obesity management*: Setting weight loss goals, focusing on portion control, developing sustainable habits rather than quick fixes, and addressing underlying factors such as stress, sleep quality, and hormonal imbalances *Behavioral changes*: Healthy snacking instead of multiple meals, nurturing healthy mindset and prioritizing health, understanding that every bite counts toward caloric need/intake, distinguishing between thirst and hunger, separating emotion (joy and sorrow, for instance) from food (avoid “stress eating”) *Physical activity*: Regular exercise (150–200 min a week), brisk walking, jogging, participating in sports, home workout routines, and walking after meals, going to gym, biking *Health monitoring*: Regular glucose level checks (As per American Diabetes Association, Patients with stable glycemic control should have their HbA₁c checked at least twice a year, while those whose treatment has changed or whose levels are above target should be tested quarterly), maintaining a health diary, consulting with a nutritionist or dietitian
Community	*Religious and community leaders*: Imams, scholars, and elders promoting health awareness during sermons and community gatherings. Teachers, school nurses, and university medical care/education to help the younger populations *Facilities*: Installing treadmills and exercise equipment in mosques, community centers (*hujras*), and other social places, hiking trails, paved sidewalks, bike tracks *Education and outreach*: Community health seminars, workshops, and fitness programs in community centers and mosques *Support groups*: Creating diabetes support groups for sharing experiences and tips, overseen by doctors in local and district headquarters/health units/hospitals *Local initiatives*: Organizing community fitness challenges (2 K/5 K), healthy cooking classes (organized by civil and nonprofit bodies), and wellness fairs/programs
District government	*Awareness Programs*: Conducting regular health awareness seminars and campaigns *Incentives for Healthy Activities*: Rewards and recognition for community members participating in health programs *Infrastructure Improvements*: Funding for playgrounds, parks, and ensuring their maintenance *Collaboration*: Engaging local NGOs, health departments, and elected officials in health initiatives *Mobile Health Clinics*: Setting up mobile clinics for remote areas to provide screenings and consultations *Lateral Screening for Depression*: Screening for commonly prevalent depression that would indirectly combat diabetes by handling stress eating
Provincial government	*Conferences and workshops*: Organizing conferences in major institutions with healthcare professionals and experts *Professional involvement*: Encouraging doctors to speak and raise awareness in communities and on national television and radio *Funding and resources*: Allocating funds to schools, colleges, and religious seminaries (*madrassas*) for gym equipment and health education materials *Curriculum changes*: Incorporating health education, physical activity, and diabetes awareness into the school curriculum *Research and data collection*: Promoting research on diabetes prevention and management, and collecting provincial-level health data
Federal government	*Taxation and regulation*: Heavily taxing companies producing sugary drinks and juices, regulating marketing of unhealthy foods *National data collection*: The National Institute of Health (NIH) collecting comprehensive data on diabetes prevalence and management *Accessible testing*: Making diabetes testing and healthcare services accessible nationwide *Legislation and policy*: Incentivizing provinces to legislate for diabetes prevention, implementing national health policies *Budget prioritization*: Prioritizing health budgets to support diabetes prevention, treatment, and management programs *Educational reforms*: Implementing federal curriculum changes to educate about diabetes, nutrition, and physical activity *Public awareness campaigns*: Launching nationwide campaigns through media, involving celebrities and influencers to promote healthy living *Information campaigns*: Launching information campaigns on mobile phones spreading awareness about diabetes

**Figure 1:**
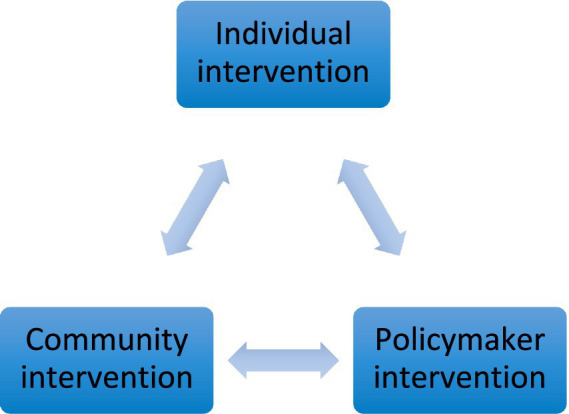
Multi-level interventions to combat diabetes in Pakistan.

Table 1 outlines three types of interventions: individual, community, and policymaker interventions. These interventions primarily work synchronously and interdependently. For example, individuals depend on community support, and communities rely on individual participation for the successful implementation of many interventions. Similarly, policymaker support is crucial for both communities and individuals to carry out some of these interventions. This relationship is illustrated in Figure 1.

Now let us briefly explain the interventions this multi-level approach may entail at various levels in the following paragraphs.

### 4.1 Individual-level interventions

At the individual level, tackling diabetes begins with lifestyle changes (21) and behavioral adjustments (20), as also supported by studies from the Diabetes Prevention Program (DPP) (Diabetes Prevention Program Group, 2002). Reducing screen time and opting for alternative activities, such as outdoor games or family engagements, can help individuals become more active, thereby reducing the risk of type-2 diabetes (22). Dietary changes are crucial as well (23): swapping sugary drinks for healthier options like sparkling water, avoiding fast foods, and opting for whole grains and non-starchy fruits and vegetables can make a significant difference. Additionally, individuals should aim to reduce their portion sizes, avoid snacking between meals to reduce caloric intake, and eat dinner earlier in the evening, followed by a walk, to help regulate insulin levels.

Effective obesity management, which includes a combination of dietary modifications, portion control, and mindful eating habits, is essential in reducing diabetes risk. Low carbohydrate and Mediterranean diets have also been shown to be effective for weight loss (24).

Physical activity plays a key role in managing insulin sensitivity, as reported in various studies (25–27). Engaging in regular exercise, such as brisk walking or jogging, for 150–200 min a week, as recommended by health guidelines, helps regulate blood sugar and manage obesity effectively (22, 28).

Health monitoring is also necessary. Individuals should regularly check their glucose levels based on risk factors and as advised by medical doctors,^4^ maintain a health diary, and consult with nutritionists to ensure they stay on track with their health goals.

### 4.2 Community-level interventions

Communities have the power to support individual efforts through collective health promotion activities (29). In Pakistan, religious and community leaders, such as *imams* and teachers, can be instrumental in raising awareness about diabetes prevention during sermons and other gatherings. For instance, imams can remind the public during Friday *khutba* (sermon) of the saying of the Prophet Muhammad (PBUH): “Verily your body has right upon you.”

Worship places such as mosques and churches, community centers and other social spaces (i.e., *hujras*) can be powerful platforms for implementing programs that promote communal health and provide other social services (30), and even more so in a culturally and religiously vibrant Muslim country like Pakistan (31). These places can be equipped with exercise equipment, such as treadmills, and can host community-driven initiatives like fitness challenges, healthy cooking classes, wellness fairs, and other community activities that encourage people to adopt healthier lifestyles (32).

Education and outreach efforts are also critical at the community level. Workshops and seminars focused on diabetes prevention, particularly in community hubs like mosques and schools, can help disseminate important information, as they have been proven effective in promoting overall wellbeing (31, 33). Additionally, establishing diabetes support groups, overseen by medical professionals, can create a supportive network where individuals share experiences and practical strategies for managing the condition—an approach that has been reported as effective in chronic disease management (34, 35).

### 4.3 Policy interventions at the district, provincial, and federal levels

Various policy approaches in LMICs have been shown to be effective in disease management and other health-related outcomes (36, 37). Pakistan itself has a track record of successful policy approaches in curtailing COVID-19 and increasing immunization rates (38).

Just as Pakistan treated COVID-19 “an existential threat” and mobilized resources and collaboration, policymakers should take similarly coordinated actions at the district, provincial, and federal levels to effectively manage the diabetes crisis.

At the district level, governments can implement awareness campaigns and offer incentives for community members to participate in health programs. Improving infrastructure by funding playgrounds and parks will encourage physical activity, while mobile health clinics can ensure that remote areas have access to screenings and consultations. It is also important to screen for common mental health conditions like depression, which can contribute to stress eating and exacerbate diabetes.

At the provincial level, governments should focus on organizing conferences and workshops with healthcare professionals to raise awareness about diabetes prevention. Allocating funds for gym equipment and health education materials in schools and religious seminaries will further encourage healthy lifestyles. Incorporating diabetes prevention into the school curriculum and promoting research on diabetes management are additional strategies to address this growing crisis.

Finally, at the federal level, broad policy changes are necessary. Islamabad can introduce taxes on sugary drinks and regulate the marketing of unhealthy foods. Nationwide diabetes testing should be made accessible, and comprehensive data collection by the National Institute of Health (NIH) will enable informed decision-making. Public awareness campaigns featuring celebrities and influencers may also help promote healthy living, while educational reforms that include lessons on nutrition and physical activity will equip future generations with the knowledge they need to prevent diabetes. Legislation that prioritizes health budgets and supports diabetes prevention programs is also essential.

## 5 Conclusion

Addressing Pakistan’s diabetes epidemic requires a coordinated, multi-level approach that engages individuals, communities, and policymakers. Through lifestyle changes including obesity management, community support, and strategic policy interventions, Pakistan can combat the rising prevalence of diabetes and work to reverse the trend. These efforts must be widespread and collaborative, ensuring that every level of society contributes to this urgent public health mission. We acknowledge that this approach is just one of many possibilities, and the policy recommendations we propose are suggestions that, if implemented, can help mitigate the diabetes crisis and improve public health outcomes.

## Footnotes

^1^ https://www.frontiersin.org/research-topics/64734/ensuring-public-health-the-active-role-of-healthcare-professionals/overview

^2^Insulin resistance refers to a condition where the body’s cells do not respond to insulin effectively (6). Insulin sensitivity, on the other hand, refers to the normal metabolic state where the body efficiently utilizes insulin (39).

^3^Genetics is also in fact a crucial factor for insulin deficiency in type 1 diabetes.

^4^According to American Diabetic Association latest guidelines (40), patients with stable glycemic control should have their HbA₁c (Hemoglobin A1c) checked at least twice a year, whereas those whose treatment has changed or whose levels are above target should be tested quarterly.
